# The Effect of Refractory Wall Emissivity on the Energy Efficiency of a Gas-Fired Steam Cracking Pilot Unit

**DOI:** 10.3390/ma14040880

**Published:** 2021-02-12

**Authors:** Stijn Vangaever, Joost Van Thielen, Jeremy Hood, John Olver, Petra Honnerovà, Geraldine J. Heynderickx, Kevin M. Van Geem

**Affiliations:** 1 Laboratory for Chemical Technology, Ghent University, Technologiepark 125, 9052 Ghent, Belgium; Stijn.Vangaever@UGent.be (S.V.); Geraldine.Heynderickx@UGent.be (G.J.H.); 2 CRESS B.V., Deltahoek 34, 4511 PA Breskens, The Netherlands; Joost.VanThielen@CRESSbv.nl; 3 Emisshield Inc., 2000 Kraft Drive VA, Blacksburg, VA 24060, USA; Jeremy.Hood@Emisshield.com (J.H.); John.Olver@Emisshield.com (J.O.); 4 New Technologies Research Centre, University of West Bohemia, Univerzitní 8, 306 14 Pilsen, Czech Republic; petrahon@ntc.zcu.cz

**Keywords:** radiative heat transfer, high emissivity coating, spectral normal emissivity, energy efficiency

## Abstract

The effect of high emissivity coatings on the radiative heat transfer in steam cracking furnaces is far from understood. To start, there is a lack of experimental data describing the emissive properties of the materials encountered in steam cracking furnaces. Therefore, spectral normal emissivity measurements are carried out, evaluating the emissive properties of refractory firebricks before and after applying a high emissivity coating at elevated temperatures. The emissive properties are enhanced significantly after applying a high emissivity coating. Pilot unit steam cracking experiments show a 5% reduction in fuel gas firing rate after applying a high emissivity coating on the refractory of the cracking cells. A parametric study, showing the effect of reactor coil and furnace wall emissive properties on the radiative heat transfer inside a tube-in-box geometry, confirms that a non-gray gas model is required to accurately model the behavior of high emissivity coatings. Even though a gray gas model suffices to capture the heat sink behavior of a reactor coil, a non-gray gas model that is able to account for the absorption and re-emission in specific bands is necessary to accurately model the benefits of applying a high emissivity coating on the furnace wall.

## 1. Introduction

Coatings modifying the emissive properties of surfaces are gaining more and more recognition in various applications including high-temperature nuclear reactors, photovoltaic solar cells, aerospace technology, military applications, the iron and steel industry, and the (petro-)chemical industry [1,2,3,4,5]. In order to assess the impact of coatings on the radiative heat transfer in all these different applications, an accurate description and comparison of both uncoated and coated material emissive properties is required. Ideally, the classical electromagnetic wave theory can be used to predict all radiative properties, including emissivity, absorptivity, and reflectivity, of any material. However, the model constants required in this theory are difficult to determine. Moreover, the surface conditions, such as surface roughness, oxide layers, and contaminants, which are all known to influence the radiative properties, are not taken into account in the electromagnetic wave theory [6]. Consequently, there is a need for an extended set of experimental data providing values for the emissive properties of a wide range of materials. However, no such database is available, with information on material emissive properties being spread over different publications and textbooks [7]. Especially for materials encountered in steam cracking furnaces, namely high temperature alloys and refractory firebricks, experimental data in scientific literature related to emissive properties are scarce.

Interest in high emissivity coatings originated in the aerospace industry. The association with spacecraft and NASA surrounded the research area with an aura of “high tech” and “mystery” [8]. Nonetheless, the underlying physical principle behind high emissivity coatings is straightforward—*it is not rocket science, except that it is*. During the atmospheric re-entry of space vehicles, friction between the space vehicle and the atmospheric gases drastically heats up the surface of the spacecraft, which is termed aero-convective heating. Since the emissive properties of a radiating surface determine the amount of heat that can be dissipated back to the surroundings, increasing the emissive properties reduces the surface temperature [9]. Emisshield licensed the high emissivity coating technology from NASA in 2001 to include all applications except for the space industry. In the last decades, high emissivity coatings started to make their way into the chemical industry. High emissivity refractory and metal coatings promise to reduce downtime, minimize coking, increase productivity, and to reduce the firebox’s overall carbon footprint by providing a uniform temperature distribution and an enhanced radiative heat transfer [10].

More recently, there has been a renewed interest in emissive properties related to daytime radiative cooling of surfaces, resulting in several publications in high-impact journals [11,12]. In this passive cooling approach, the emissive properties of a surface are tuned to allow the surface to cool below the ambient air temperature, even under direct sunlight and without the need for electricity [4]. In both examples of aerospace and daytime radiative cooling, by tuning the emissive properties, the “hot surface” emits radiation in a specific wavelength range thereby using the cold darkness of the Universe as a renewable thermodynamic source [13,14].

In the last few decades, various patents have been granted to industrial players that started to explore the possibility to use high emissivity coatings in the chemical, iron, steel, and glass industry [15,16]. These industries heavily depend on large industrial furnaces where radiation is the dominant heat transfer mechanism. However, there is a fundamental difference in the role of coatings between, on the one hand, industrial furnaces and, on the other, aerospace applications or radiative cooling. In industrial furnaces, the temperature difference between the emitting and receiving body is less pronounced, making it harder to select a wavelength range where emission or reflection should be optimized. For daytime radiative cooling, in contrast, it is clearly beneficial to have high emissive properties in the “sky window”, between 8 and 13 µm, where the atmosphere is nearly transparent, allowing the surface to emit thermal radiation to outer space. Additionally, the surface has to reflect as much radiation as possible in the solar spectrum, situated between 0.3 and 2.5 µm, for daytime radiative cooling applications [17]. For gas-fired furnaces, almost all radiative heat transfer occurs in the thermal infrared wavelength range, 0.8–25 µm [18]. Hence, the industrially used high emissivity coatings maximize the emissive properties over the entire wavelength range. With this approach, it becomes more likely that the energy emitted by the flue gas is absorbed and re-emitted by the furnace wall in a clear window of the flue gas, thereby heating the furnace load.

Various researchers have studied the effect of surface emissive properties on the energy efficiency of industrial furnaces. Early research stressed the importance of both clear and absorbing wavelength bands when modelling radiative heat transfer in the presence of flue gases in order to accurately evaluate the effect of the furnace wall emissivity [19,20]. Ward et al. developed a spectral model to predict the transient performance of a metal reheating furnace using a zoning method. Increasing the refractory furnace wall emissivity resulted in energy savings of approximately 3% [21,22]. Heynderickx et al. and Stefanidis et al. performed three-dimensional Computational Fluid Dynamics simulations of a naphtha cracking furnace with varying furnace wall emissivity [5,23,24,25]. Adams et al. performed similar Computational Fluid Dynamics simulations to estimate how high emissivity coatings can reduce the fuel firing rate in combustion-heated process furnaces [26]. Spectral radiative heat transfer simulations show that increasing the refractory wall emissivity from 0.4 to 0.9 resulted in a 1.5% increase in furnace efficiency. In the same work, energy savings observed in industrial furnaces, after applying a high emissivity coating, are reported ranging from 8% for glass melting furnaces to 5 and 6% for ethylene dichloride and steam cracking furnaces, respectively. More recently, Yi et al. developed an analytical expression to study the effect of furnace wall emissivity on the heating process of a steel slab furnace, stating that high emissivity coatings might even have a negative impact, hindering the radiative heat transfer [27].

In parallel to these numerical studies, various experimental studies have been conducted to assess the impact of high emissivity coatings on radiative heat transfer in furnaces. Hellander et al. reported energy saving exceeding 17% for an annealing furnace [28]. Benko et al. reported experimental energy savings in the range of 4.8–6.2% and 3.8–4.4% for natural gas- fired and oil-fired furnaces, respectively [29]. Svantner et al. investigated the influence of furnace wall emissivity on steel charge heating, thereby stressing the importance of the emissive properties in different wavelength bands [30]. The significant spread in reported energy savings, shown in Table 1, makes it challenging to predict the exact effect of applying a high emissivity coating on the energy efficiency of a specific furnace.

This work aims to develop a more straightforward simulation framework to assess the effect of reactor coil and furnace wall emissive properties on the radiative heat transfer in gas-fired furnaces. Recent scientific work confirms that in some contexts it is sufficient to perform radiative heat transfer simulations of simplified geometries. Often, the numerical study of simplified geometries suffices to give insight in radiative heat transfer characteristics. When time consuming Computational Fluid Dynamics simulations of more complex geometries are avoided, more elaborate parametric studies can be performed [22,31,32]. For example, Hu et al. revisited Hottel’s zoning method for the firebox modelling of a steam cracking furnace and confirmed that this computationally less expensive approach provides reliable input for the first design steps of new furnaces or for the optimization of existing furnaces [33].

The subsequent section emphasizes the different aspects of “emissivity” before gathering experimental data describing the emissive properties of uncoated and refractory firebricks. In the next step, the effect of a refractory coating on the firing rate of a steam cracking pilot unit is tested experimentally. Additionally, a parametric study is performed to evaluate the effect of the boundary wall emissive properties on the radiative heat transfer in a so-called tube-in-box geometry.

## 2. Terminology

The three fundamental modes of heat transfer are conduction, convection, and radiation. Both conduction and convection require the presence of a medium in order to transfer energy. Thermal radiation is transferred by electromagnetic waves, which implies that no participating medium is required. Another distinguishing feature between conduction and convection, on the one hand, and thermal radiation, on the other, is the difference in their temperature dependency [34]. In most applications, conductive and convective heat transfer rates are linearly proportional to a temperature difference [34]. In contrast, the Stefan-Boltzmann equation for a blackbody radiator expresses that the total emissive power of a blackbody, $E_{b}\left(T\right)$, has a biquadratic relation to the blackbody temperature T:

(1) $$E_{b}\left(T\right)=\sigma T^{4}$$

where σ is the Stefan-Boltzmann constant. This biquadratic relation indicates that radiation will be the predominant mode of heat transfer in high-temperature applications. In reality, no material behaves as a perfect blackbody. When an electromagnetic ray strikes an opaque material surface, the ray is either reflected or absorbed. Kirchhoff’s law of thermal radiation states that a surface at thermal equilibrium emits the same amount of energy as it absorbs. This statement, just like Kirchhoff’s laws for electric circuits, is just a rephrasing of the universal law of conservation of energy. The most general definition of “emissivity” describes emissivity as the ratio of the actual material surface irradiance to the irradiance of a perfect blackbody. The emissivity, in other words, defines the deviation of surface irradiance from a perfect blackbody, resulting in a value between zero and one, with one indicating blackbody behavior. Throughout this work the term “emissivity” is avoided as much as possible, even though it is frequently used throughout scientific literature and standard textbooks. For example, in the introduction of this paper, the term “emissive properties” was used to replace the general term “emissivity”. When discussing and comparing emissive properties, it is essential to clarify which emissive property is being referred to. For this reason, the next section is dedicated to introducing the different aspects of “emissivity”.

**Figure 1:**
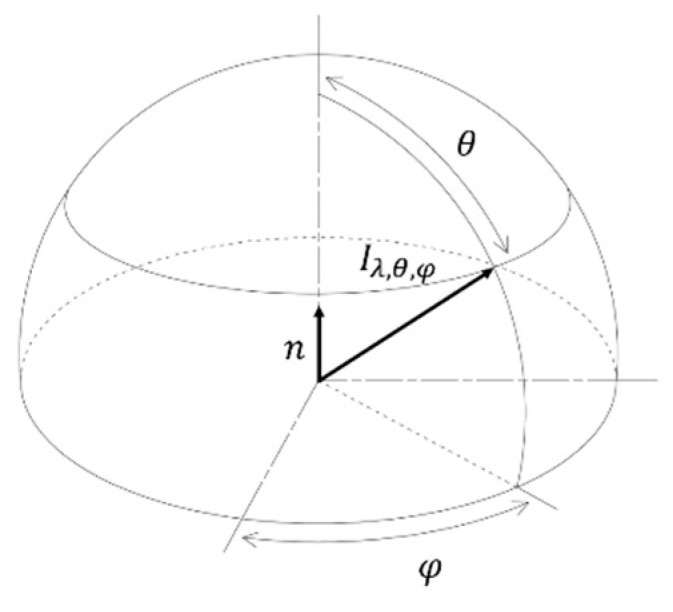
Spectral directional irradiance and polar coordinates as related to a unit hemisphere.

The most fundamental radiative property for emission from a surface is the *spectral directional* emissivity defined as:

(2) $$\varepsilon_{\lambda ,\theta ,\varphi }\left(\lambda ,\theta ,\varphi ,T\right)=\frac{I_{\lambda ,\theta ,\varphi }\left(\lambda ,\theta ,\varphi ,T\right)}{I_{\lambda }^{B}\left(\lambda ,T\right)}$$

which compares the actual *spectral directional* irradiance, $I_{\lambda ,\theta ,\varphi }(\lambda ,\theta ,\varphi ,T$), as shown in Figure 1, to that of an ideal blackbody, $I_{\lambda }^{B}\left(\lambda ,T\right)$, at the same temperature and under the same geometrical and spectral conditions [34].

*Spectral directional* emissivity does not only depend on the temperature T, wavelength λ, polar angle θ, and azimuth angle φ, but also on the surface roughness and on the chemical composition of the surface. These last two variables are harder to quantify and are locally varying surface properties. In order to compare experimentally measured emissive properties of different samples, the National Institute of Standards and Technology (NIST) proposed to use the term *emissivity* for optically smooth and homogeneous materials and to use the term *emittance* for rough and contaminated surfaces [35]. However, these terms are not generally used. In this work, the term *apparent* emissivity is introduced to define the experimentally observed emissivity of real surfaces. *Intrinsic* emissivity is used to refer to perfectly smooth materials [36,37].

The most fundamental emissive property, the *spectral directional* emissivity, Equation (2), is hardly ever measured experimentally since it depends on both wavelength and polar coordinates. A lot of expensive, moving instrumental parts are required to scan the *spectral directional* emissivity experimentally. The most recent experimental units measure the *spectral normal* emissivity [38,39,40]. In this case, the irradiance is captured by a detector right in front of the sample. In other words, the detector is placed in a way that both polar coordinates become zero, i.e., detector placed perpendicular to the surface. Accounting for Equation (2), the *spectral normal* emissivity, $\varepsilon_{\lambda ,n}\left(\lambda ,T\right)$, can be defined as:

(3) $$\varepsilon_{\lambda ,n}\left(\lambda ,T\right)=\varepsilon_{\lambda ,\theta =0{}^{\circ},\varphi =0{}^{\circ}}\left(\lambda ,T\right)=\frac{I_{\lambda ,n}\left(\lambda ,T\right)}{I_{\lambda }^{B}\left(\lambda ,T\right)}$$

The experimentally measured *spectral normal* emissivity is thus an *apparent* value of an actual surface. However, the *spectral normal* emissivity approaches the *intrinsic* value for very smooth surfaces.

Integrating the *spectral normal* emissivity, Equation (3), over the entire wavelength range using the blackbody irradiance, $I_{\lambda }^{B}\left(\lambda ,T\right)$, governed by Planck’s law, as a weight function gives the *total normal* emissivity, $\varepsilon_{tot,n}\left(T\right)$, at a given temperature:

(4) $$\varepsilon_{tot,n}\left(T\right)=\frac{\int_{0}^{+\infty }I_{\lambda ,n}\left(\lambda ,T\right)d\lambda }{\int_{0}^{+\infty }I_{\lambda }^{B}\left(\lambda ,T\right)d\lambda }=\frac{\int_{0}^{+\infty }\varepsilon_{\lambda ,n}\left(\lambda ,T\right).I_{\lambda }^{B}\left(\lambda ,T\right)d\lambda }{\int_{0}^{+\infty }I_{\lambda }^{B}\left(\lambda ,T\right)d\lambda }$$

Since most *spectral normal* emissivity, Equation (3), measurement devices only measure part of the entire electromagnetic spectrum, the term *band normal* emissivity, Equation (5), is typically introduced to emphasize this wavelength band dependency. The *band normal* emissivity for a given wavelength band $\left[\lambda_{1},\lambda_{2}\right]$ at a temperature, T, is given by the following definition:

(5) $$\varepsilon_{\left[\lambda_{1},\lambda_{2}\right],n}\left(T\right)=\frac{\int_{\lambda_{1}}^{\lambda_{2}}I_{\lambda ,n}\left(\lambda ,T\right)d\lambda }{\int_{\lambda_{1}}^{\lambda_{2}}I_{\lambda }^{B}\left(\lambda ,T\right)d\lambda }=\frac{\int_{\lambda_{1}}^{\lambda_{2}}\varepsilon_{\lambda ,n}\left(\lambda ,T\right).I_{\lambda }^{B}\left(\lambda ,T\right)d\lambda }{\int_{\lambda_{1}}^{\lambda_{2}}I_{\lambda }^{B}\left(\lambda ,T\right)d\lambda }$$

When the *band normal* emissivity, Equation (5), is evaluated over the entire experimental wavelength range, the concepts *total normal* emissivity, Equation (4), and *band normal* emissivity, Equation (5), are often used interchangeably. This is considered to be a valid approximation when most of the radiation is emitted in the experimentally covered wavelength range $\left[\lambda_{1},\lambda_{2}\right]$.

When the *spectral directional* emissivity, Equation (2), is integrated over both the entire hemisphere and over the entire wavelength range using the blackbody irradiance as a weight function, the *total hemispherical* emissivity is calculated:

(6) $$\varepsilon_{tot}\left(T\right)=\frac{\int_{0}^{+\infty }\int_{0}^{2\pi }\int_{0}^{\pi /2}\varepsilon_{\lambda ,\theta ,\varphi }\left(\lambda ,\theta ,\varphi ,T\right).I_{\lambda }^{B}\left(\lambda ,T\right)\cos\left(\theta \right)\sin\left(\theta \right)d\theta \;d\varphi \;d\lambda }{\int_{0}^{+\infty }\int_{0}^{2\pi }\int_{0}^{\pi /2}I_{\lambda }^{B}\left(\lambda ,T\right)\cos\left(\theta \right)\sin\left(\theta \right)d\theta \;d\varphi \;d\lambda }$$

The *total hemispherical* emissivity, Equation (6), is the most frequently reported emissive property and can easily be measured using calorimetric emissivity measurement devices [2,41]. The American Society for Testing and Materials (ASTM) has even developed a standard test method, ASTM C835-06, for the *total hemispherical* emissivity of surfaces up to 1673 K [42].

## 3. Methods and Procedures

### 3.1. Spectral Normal Emissivity Measurements

Various experimental units have been developed to measure the emissive properties of material surfaces. The main two categories include *calorimetric* measurement devices where the total hemispherical emissivity is measured and *indirect radiometric* methods. The *indirect radiometric* methods rely on Kirchhoff’s law of thermal radiation to calculate the emissive properties based on reflected radiation. However, *calorimetric* measurement devices and *indirect radiometric* methods are being replaced by more advanced *direct radiometric* methods, where wavelength dependent emissive properties can be measured directly at high temperatures. In this case, the advantage of *calorimetric* measurement devices, namely operation at high temperatures, is combined with measuring the wavelength dependency, which is the main benefit of using *radiometric* methods. The *direct radiometric* units designed to measure the spectral normal emissivity mainly consist of a reference source of radiation, an FTIR spectrometer (for example Nicolet series from Thermo Fisher Scientific Inc., Waltham, MA, USA), a sample heating method, and a surface temperature detector [43].

**Figure 2:**
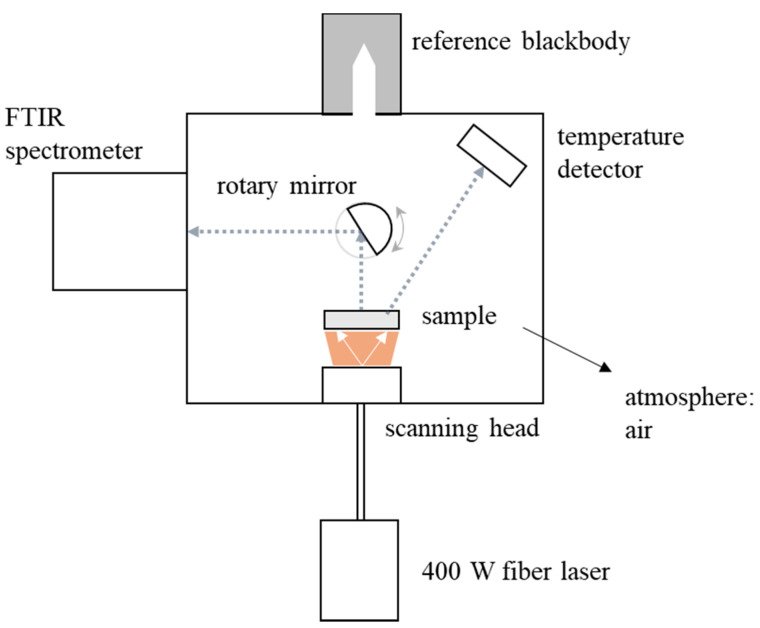
Schematic overview of the spectral normal emissivity measurement device at the New Technologies Research Center at the University of West Bohemia.

The spectral normal emissivity measurement unit of the New Technologies Research Center at the University of West Bohemia, shown in Figure 2, is described in detail by Honnerova et al. [38]. A sample is heated in dry air using a 400 W fiber laser [44] while the surface temperature is controlled using a calibrated infrared camera as temperature detector [45]. The computer-controlled calibrated laboratory blackbody allows the unit to perform measurements up to 1255 K. The FTIR spectrometer allows measurements in the wavelength range from 1.38–26 µm, with the lower wavelength limit being determined by the radiation source temperature. Disk-shaped samples of the studied (un)coated material with a 25 mm diameter and a 5 mm thickness are used to perform the measurements. Refractory samples tend to be porous and are designed to have a low thermal conductivity. This insulating nature of refractory complicates laser heating, limiting the maximum operating temperature to 873 K.

### 3.2. Pilot Steam Cracker

**Figure 3:**
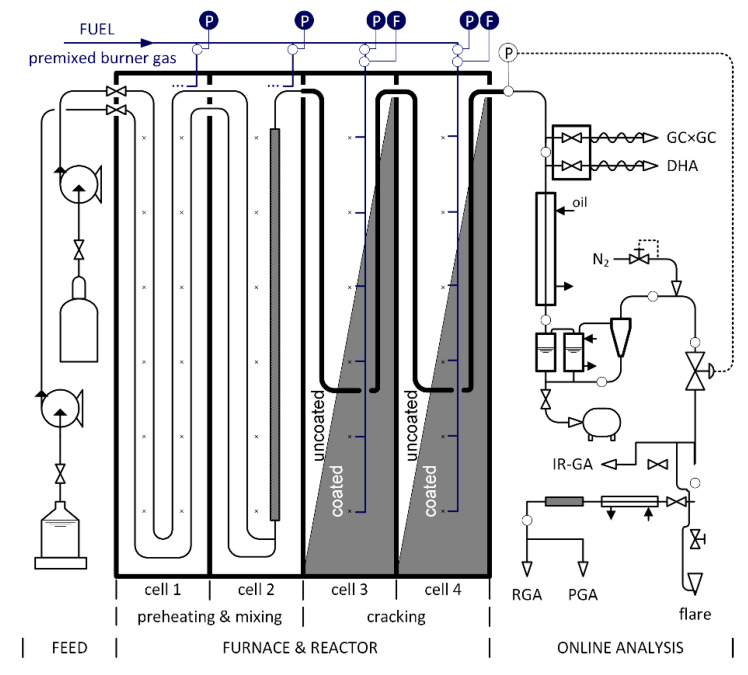
Visual representation of the steam cracking pilot unit at the Laboratory for Chemical Technology at Ghent University. The pilot unit id divided into a feed section, a furnace-reactor section, and an online analysis section [47].

The Ghent University steam cracking unit is used to gather data on a pilot scale. For a detailed description of this reactive unit, shown in Figure 3, including all the analytical equipment, the reader is referred to previous work [46,47].

The feed section consists of different pumps and mass flow controllers so a wide variety of both gaseous and liquid hydrocarbon feedstocks can be fed to the reactor. The furnace consists of four separate cells for which the temperature can be controlled individually. Twelve natural gas-fired wall burners in each cell allow the operator to impose any required temperature or heat flux profile along the reactor tube length. The pilot unit can be used to test the effect of different feedstock mixtures, reactor materials, reactor designs, or firebox modifications on the steam cracking process.

The described pilot unit is used to test the effect of applying a high emissivity coating on the refractory wall on the energy efficiency of a gas-fired furnace. The following experimental procedure is used before and after applying a high emissivity coating on the refractory wall.

In the first two cells, see Figure 3, 10 kg/h of propane is mixed with 4 kg/h of dilution steam and preheated to the desired coil inlet temperature of 917 K. An advanced rifled reactor geometry made of a high-temperature aluminum containing alloy is installed in the next two cells, the cracking cells where the endothermic reactions take place [48]. At the outlet of the last cell a coil outlet temperature of 1111 K is maintained to guarantee the same cracking severity for each experiment, corresponding to 90% propane conversion.

For every experiment, the cracking cycle lasts 6 h before the experiment is halted and the reactor is decoked. The experimental procedure is repeated two times to establish a base case with uncoated furnace walls. Next, the two cracking cells are coated with a high emissivity coating and the experimental procedure is repeated twice. The operating conditions for a steam cracking cycle including pre-sulfidation, the cracking step, and decoking are given in Table 2. The operating temperature mentioned for cracking cell 3 and 4, see Figure 3, is imposed at the outlet of that cell.

Differential pressure transmitters are installed to measure the premixed burner gas flow rate to the two cracking cells. Additionally, the total natural gas flow rate to the entire furnace is monitored. Several thermocouples are installed to measure the flue gas temperature, the process gas temperature, the tube metal wall temperature, and the refractory wall temperature. The refractory wall temperature is measured both inside and outside the furnace. These measurements help the operator to close the energy balance.

The composition of the high emissivity coating used in the spectral normal emissivity measurements and on the pilot-scale furnace is proprietary information. The reader is referred to the corresponding patent for more detail [16].

### 3.3. Radiative Heat Transfer Modelling

**Figure 4:**
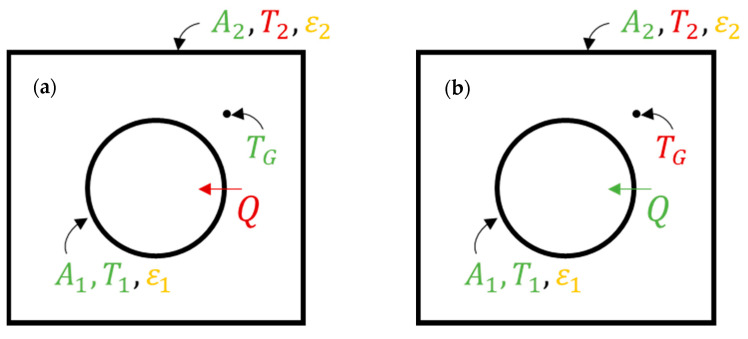
Tube-in-box model where (**a**) a constant gas phase temperature, $T_{G}$, is assumed for varying tube, $\varepsilon_{1}$, and adiabatic surface, $\varepsilon_{2}$, emissive properties and (**b**) the gas phase temperature, $T_{G}$, is adjusted to get the same heat flux, Q, again for varying $\varepsilon_{1}$ and $\varepsilon_{2}$.

A simplified geometry, based on a horizontal cross-section of one of the pilot unit cracking cells, is studied to assess the effect of boundary wall emissive properties on radiative heat transfer. This so-called tube-in-box model, shown in Figure 4, consists of a central cylindrical surface, acting as a heat sink, and an enclosing adiabatic refractory surface. The enclosing adiabatic square-shaped box has a length of 0.380 m. A tube with a diameter of 0.103 m, equivalent to the pilot unit heat sink dimensions, is positioned at the center of the box. A perfectly stirred flue gas with a uniform gas temperature, $T_{G}$, is situated between the adiabatic enclosure and the central heat sink. The flue gas is composed of CO_2_, H_2_O and N_2_ in the stoichiometric combustion ratio of methane in air. Only radiative heat transfer is accounted for, neglecting convection and conduction from the perfectly stirred gas to the surrounding surfaces.

Radiative heat transfer from the hot flue gas to the central heat sink will be modeled with varying emissive properties of both the heat sink, $\varepsilon_{1}$, and the furnace refractory wall, $\varepsilon_{2}$. The geometry is determined by the surface areas, $A_{1}$ and $A_{2}$ (corresponding to a pseudo two-dimensional representation with a height of 1 m). The central heat sink temperature, $T_{1}$, is kept constant and corresponds with the averaged experimentally observed tube metal temperature. Initially, the gas phase temperature, $T_{G}$, is kept constant resulting in an unknown adiabatic enclosing wall temperature, $T_{2}$, and an unknown heat flux, Q, to the central heat sink. The initial case is visualized in Figure 4a. In a second simulation, shown in Figure 4b, the gas phase temperature, $T_{G}$, is varied to obtain a constant heat flux, Q, for varying wall emissive properties. The two described radiative heat transfer case studies will initially be solved with a non-gray gas assumption. Later, this solution will be compared to the results obtained when using a simplified gray gas approach.

The “exchange areas approach” was first introduced by Hottel and Sarofim in their zonal method for radiative heat transfer [49]. The total surface-to-surface exchange areas, $\bar{S_{i}S_{j}}\prime$, with a participating non-gray gas and the total gas-to-surface exchange areas, $\bar{GS_{i}}\prime$, are derived in the supporting information for the gray-plus-clear gas solution to the tube-in-box geometry. In the gray-plus-clear gas model, the weighted sum of gray gases model is reduced to the combination of one lumped absorption band, where the total exchange areas derived for a gray gas are assumed to be applicable, and one clear band, where the gas absorbs no radiation [50].

$${\bar{S_{1}S_{2}}}^{\prime }=\left(1-a\right)\frac{A_{1}\varepsilon_{1}\varepsilon_{2}}{1-\rho_{2}\left(1-\varepsilon_{1}A_{1}/A_{2}\right)}+a\frac{A_{1}\varepsilon_{1}\varepsilon_{2}}{D}$$

(7) $${\bar{GS_{1}}}^{\prime }=\frac{A_{1}\varepsilon_{1}\varepsilon_{G}\left(1/\tau +\rho_{2}A_{1}/A_{2}\right)}{D}$$

$$\mathrm{with}\;D=1/\tau -\rho_{2}\left[1-\frac{A_{1}}{A_{2}}\left(1-\tau \rho_{1}\right)\right]$$

The coefficient a represents the fraction of the total energy transferred in the non-clear band, where the gas phase emissivity, $\varepsilon_{G}$, and transmissivity, $\tau =1-\varepsilon_{G}$, for a gray gas are valid. Opaque surfaces do not transmit radiation, resulting in the following expression for the reflectivity of a surface i, $\rho_{i}=1-\varepsilon_{i}$. The enclosing surface is assumed to be adiabatic, which indicates that all incoming energy will have to be redirected:

(8) $$Q_{tot}=\left({\bar{GS_{1}}}^{\prime }+\frac{1}{\frac{1}{{\bar{GS_{1}}}^{\prime }}+\frac{1}{{\bar{S_{1}S_{2}}}^{\prime }}}\right)\left(E_{g}-E_{1}\right)$$

The exchange areas in Hottel’s zoning method are often reduced to a gray gas implementation, solving the radiative heat transfer in one absorption band only. In that case, the total surface-to-surface exchange areas and the total gas-to-surface exchange areas for the tube-in-box model are reduced to:

$$\bar{S_{1}S_{2}}=\frac{A_{1}\varepsilon_{1}\varepsilon_{2}}{D}$$

(9) $$\bar{GS_{1}}=\frac{A_{1}\varepsilon_{1}\varepsilon_{G}\left(1/\tau +\rho_{2}A_{1}/A_{2}\right)\;}{D}$$

$$\mathrm{with}\;D=1/\tau -\rho_{2}\left[1-\frac{A_{1}}{A_{2}}\left(1-\tau \rho_{1}\right)\right]$$

Finally, substitution of the total surface-to-surface and gas-to-surface exchange areas in Equation (8) gives the following analytic expression for the total heat flux in the presence of a gray gas:

(10) $$Q_{tot}=A_{1}\frac{\varepsilon_{1}\varepsilon_{G}\left[1+\frac{A_{1}}{A_{2}}\left(1-\varepsilon_{G}\right)\right]}{\varepsilon_{G}+\frac{A_{1}}{A_{2}}\left(1-\varepsilon_{G}\right)\left[\varepsilon_{1}+\varepsilon_{G}\left(1-\varepsilon_{1}\right)\right]}\left(E_{g}-E_{1}\right)$$

The correlation in Equation (10) is frequently relied upon in radiative heat transfer simulations but fails to capture the effect of the emissive properties of the surrounding adiabatic wall, $\varepsilon_{2}$, as discussed in more detail later in this work [20,27].

## 4. Results and Discussion

### 4.1. Spectral Normal Emissivity Measurements

**Figure 5:**
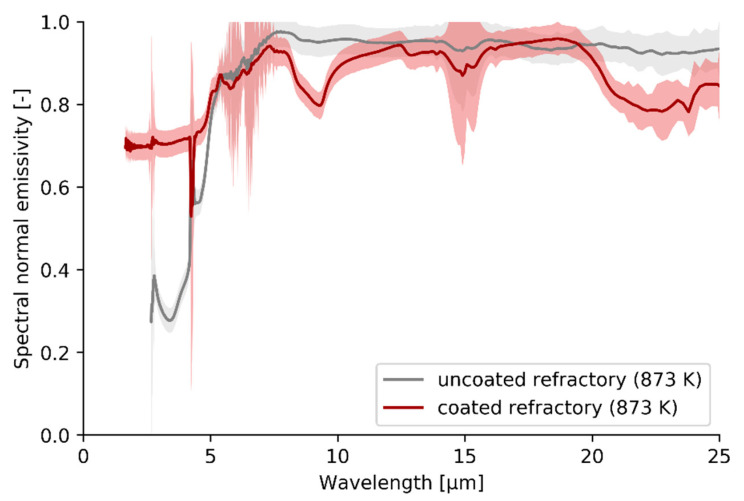
Spectral normal emissivity measurement results for uncoated refractory and refractory coated with a high emissivity coating. The uncertainty of the spectral normal emissivity results is indicated by the transparent bands (New Technologies Research Center, University of West Bohemia).

The spectral normal emissivity measurement results of the pilot unit refractory material before and after a high emissivity coating is applied are shown in Figure 5. Since the spectral normal emissivity measurement apparatus at the New Technologies Research Center operates in an air atmosphere, the experimental uncertainty, also shown in Figure 5, is higher in wavelength bands where atmospheric gases interfere along the optical path [51]. At the shortest wavelengths, below 5 µm, the spectral normal emissivity of the high emissivity coating is significantly higher compared to the uncoated refractory results. At longer wavelengths, the uncoated sample slightly outperforms the coated sample. However, at high temperatures, e.g., in steam cracking furnaces, proportionally more energy is emitted at lower wavelengths in accordance with Planck’s law of thermal radiation. The step-like spectral normal emissivity reported for the refractory substrate matches the limited data available for silica-alumina firebricks [52,53].

A more quantitative comparison between both spectral normal emissivity measurements is possible by introducing the *band normal* emissivity. The *band normal* emissivity is calculated by integrating the *spectral normal* emissivity over the entire experimental range while accounting for the spectral density at a certain temperature specified by Planck’s law, Equation (5). Since most of the thermal radiative heat transfer takes place in the experimentally covered wavelength range, the *band normal* emissivity approximates the more widely reported *total normal* emissivity, Equation (4). Weighted integration, accounting for the wavelengths where proportionally more energy is emitted, yields a spectral normal emissivity of 0.79 for the high emissivity coating compared to 0.67 for the uncoated refractory at 873 K; the coating emissive properties exceed those of the base substrate.

**Figure 6:**
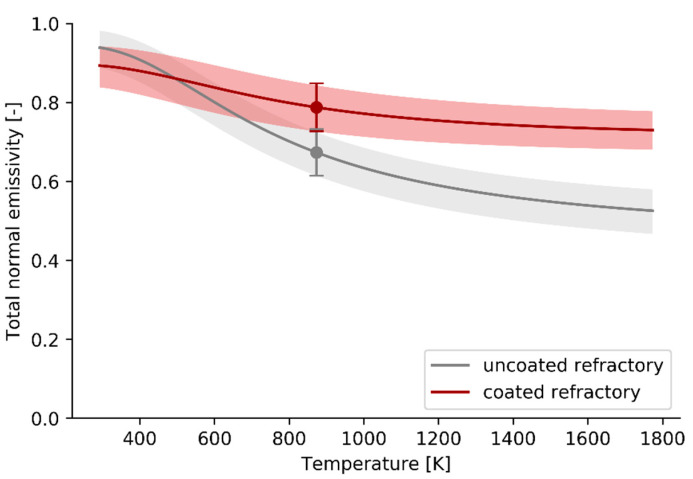
Total normal emissivity results for uncoated refractory and coated refractory calculated based on the spectral normal emissivity measurements performed at 873 K.

If the *spectral normal* firebrick emissivity measurement results, shown in Figure 5, are assumed to be temperature independent, the *total normal* emissivity can be extrapolated to a wider temperature range as shown in Figure 6.

The total normal emissivity results confirm that the coating outperforms the base substrate at higher temperatures when proportionally more radiation is emitted in the short wavelength region where the spectral normal emissivity is relatively low for the base substrate. Due to the poor thermal conductivity of the refractory substrate only measurements up to 873 K are performed. The extrapolation assumes that the spectral normal emissivity is temperature independent.

**Figure 7:**
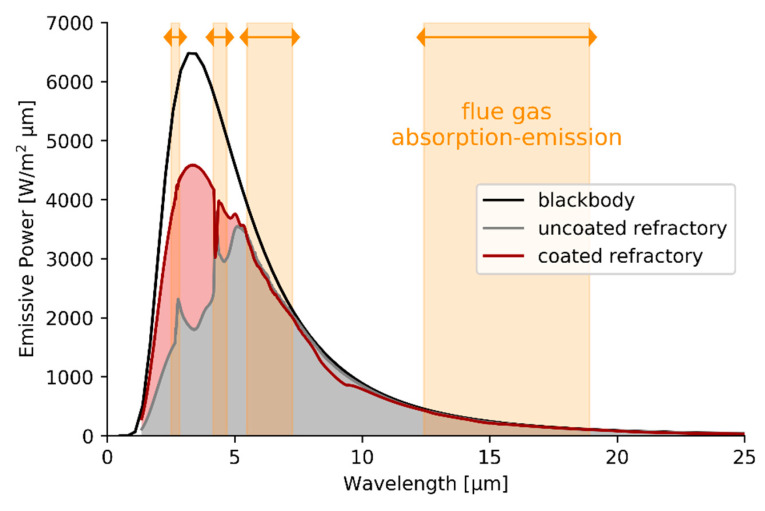
Emissive power for a perfect blackbody, uncoated refractory, and coated refractory at 873 K. The flue gas absorption-emission bands are highlighted.

Figure 7 shows the emissive power in accordance with Planck’s law for a perfect blackbody and both an uncoated and a coated firebrick. The emissive power spectrum is yet another representation based on the spectral normal emissivity measurement results previously shown in Figure 7. The flue gas absorption-emission bands are introduced for the first time in this work. The band upper and lower limits for each band have been taken from Zhang et al. [18]. The participating species, water, and carbon dioxide, present in the flue gas of gas-fired furnaces, emit and absorb radiation in these wavelength ranges. These wavelength ranges are defined by the rotational and vibrational molecular frequencies of the participating species. Based on the emissive power spectra at 873 K, shown in Figure 7, the uncoated firebrick emits 67% of its total emitted radiation in the clear windows in contrast to the coated refractory where 74% the total emitted radiation ends up in the clear windows. At a more relevant temperature for combustion applications of 1573 K, 85% of the emitted radiation ends up in the clear widow for uncoated refractory. Applying a high emissivity coating on the firebrick increases the fraction of radiation that is emitted in the clear window to 88%. The approach of visualizing emitted radiation in specific windows highlights that there are energy savings are possible when applying a high emissivity coating. Radiation can be emitted from the furnace wall to the heat sink, i.e., reactor coil, in a clear window where it cannot be re-absorbed by the gas phase.

An alternative way of designing coatings would be aimed at maximizing the emissive properties only in the clear windows and to selectively tune the emissive properties to reflect radiation in the absorption bands. This alternative approach would work similarly to coatings used in daytime radiative cooling applications as explained in the introduction, where emissive properties are enhanced in predefined regions. This way, all radiation, 100% in theory, would be emitted in the clear windows. However, one should not forget that the combustion gases are providing the necessary energy in gas-fired furnaces, not the refractory wall. The theoretical limiting case of emitting all radiation in clear windows also implies that all radiation coming from the flue gas in absorption-emission bands will be reflected in wavelength regions where it can easily be re-absorbed by the flue gases. To avoid this phenomenon, high emissivity coatings typically increase the emissive properties over the entire wavelength range, maximizing the possibility to absorb radiation in the absorption bands and re-emit the energy in a clear window. Here the role of “emissivity” is not only regarded as the raw emissive power of a body at a specific temperature, but also as a way to absorb and re-emit radiation.

In order to properly evaluate the effect of coating the refractory wall on the energy re-distribution in a gas-fired furnace, pilot unit experiments are performed and discussed in the following section. In a next step, radiative heat transfer simulations are performed in a tube-in-box geometry. In contrast to the percentages derived based on Figure 7, not only emission but also reflection and absorption followed by re-emission of the participating surfaces will be modelled accounting for the surrounding gas. Initially, a non-gray gas model will be used to capture the gas phase absorption-emission characteristics. Next, a simplified gas model will be implemented. The subsequent discussion focuses on why only a non-gray gas implementation is able to quantify the effect of applying a high emissivity coating on the refractory wall and what this tells us about the role of high emissivity coatings on radiative heat transfer.

### 4.2. Pilot Scale Experiments

**Figure 8:**
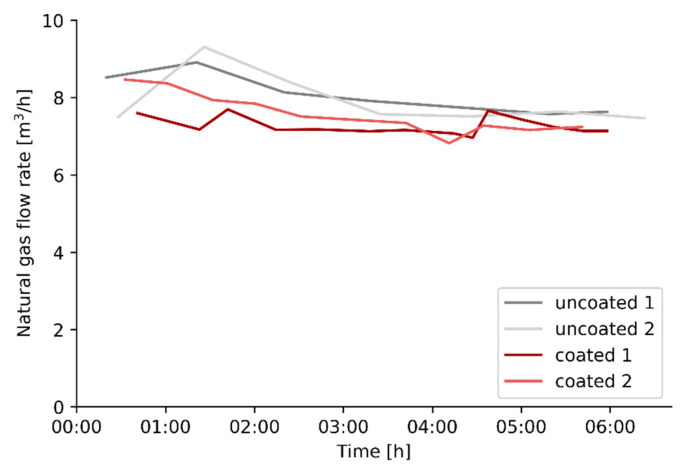
Natural gas flow rate to the entire pilot unit as a function of time for the different experiments.

With the spectral normal emissivity results of (un)coated refractory, shown in Figure 5, in mind, the effect of the same coating on the firing rate of a pilot steam cracker will be evaluated experimentally. Four experiments, two reference experiments and two experiments after applying a high emissivity coating on the refractory wall of the cracking cells, are performed with the same operating conditions and cracking severity as specified in Table 2. The fuel, i.e., natural gas, consumption of the steam cracking pilot unit before and after the high emissivity coating is applied, is shown in Figure 8. In all experiments, the natural gas consumption decreases over time to reach a steady-state after almost 6 h of cracking. The decrease can be attributed to the fact that the entire furnace initially needs to heat-up and find an equilibrium with the surrounding environment.

The fuel consumption at steady state is summarized in Table 3. The application of the selected high emissivity coating results in a decrease in fuel firing rate of 4.8%, based on averaged values for the uncoated and coated runs when the system has reached a steady state.

**Figure 9:**
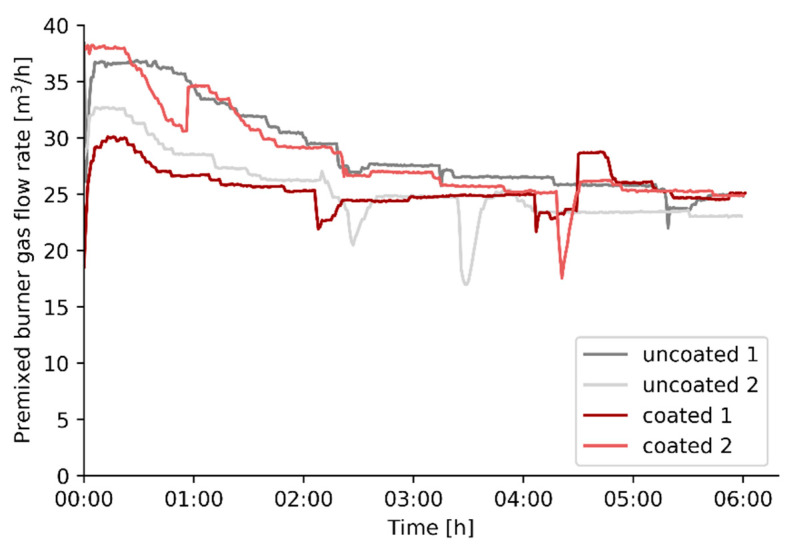
Premixed burner gas flow rate to cells 3 and 4 combined versus time for the different experiments.

Figure 9 shows the total flow rate of premixed burner gas, that is natural gas mixed with combustion air, going towards cells 3 and 4 of the pilot unit, where the cracking reactions take place in the suspended coil. No real energy savings are observed here. This implies that the reduced firing is mostly limited to the initial two preheating cells. However, there is no coating present in the initial two preheating cells and the process gas enters the cracking cells with the exact same temperature, indicating that the same amount of energy has been absorbed by the process gas in the preheating cells.

**Figure 10:**
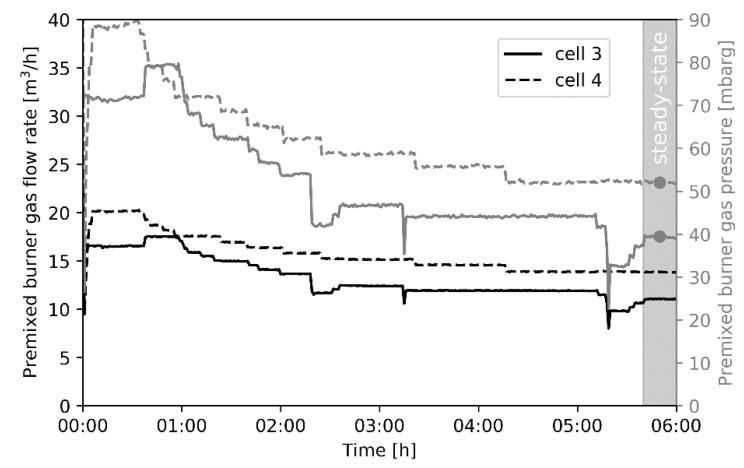
Premixed burner gas flow rate to cell 3 (dashed line) and cell 4 (full line) individually (left) and premixed burner gas pressure (right) for the first uncoated experiment (uncoated 1).

In contrast to the premixed burner gas flow meters, which are only installed in the cracking cells, the premixed burner gas pressure is measured for every cell. The premixed burner gas pressure is correlated to the premixed burner gas flow rate by Bernoulli’s equation and provides qualitative information about the distribution of premixed burner gas to the different cells, even for the preheating cells where no flow meters are present. The correlation between premixed burner gas flow rate and premixed burner gas pressure is visualized in Figure 10 for cells 3 and 4 individually.

**Figure 11:**
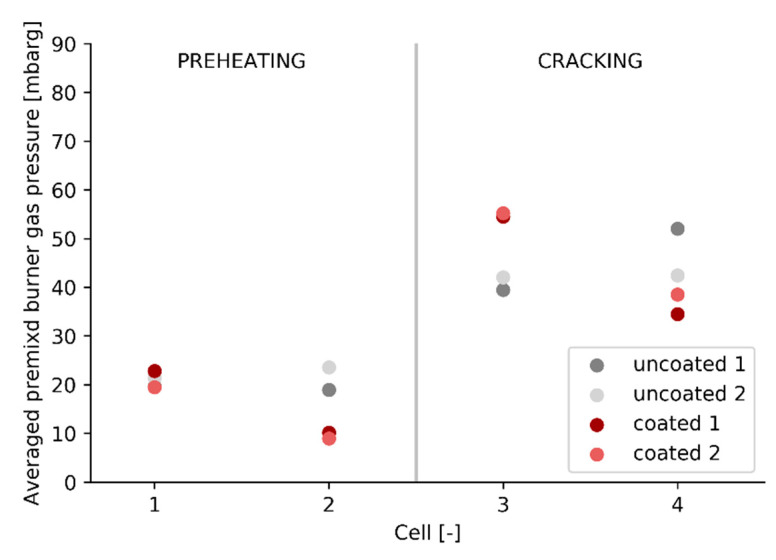
Premixed burner gas pressure measured before every cell for the different experiments. The premixed burner gas pressure is taken at the end of the experiment when the process has reached steady state.

The premixed burner gas pressure for each experiment at the end of the cracking cycle, when the system has reached steady state, is shown in Figure 11, for all four cells. The averaged burner gas pressure, and thus the premixed burner gas flow, increases in cell 3 and decreases in cell 2 after the high emissivity coating is applied. This suggests that for the coated experiments, the energy from the third cell is partly used to heat the adjacent preheating cell 2. This phenomenon can be explained by the geometric nature of the experimental unit. The cells are located directly next to each other which makes that a cell can overcompensate the burner gas flow rate for one of its adjacent cells. This explains why energy savings are visible when looking at the total natural gas flow rate, Figure 8, but not apparent when comparing the premixed burner gas flow rate to the cracking cells before and after applying a high emissivity coating, Figure 9. Based on the premixed burner gas pressure at steady state, shown in Figure 11, the second preheating cell, cell 2, is underperforming. After the coating is applied in cells 3 and 4, the process control loop of the pilot plant operates in a suboptimal region where cell 3 is overcompensating for cell 2.

The pilot scale experiments show a fuel efficiency increase of 4.8%, based on the firing rate for the uncoated and coated runs when the system has reached a steady state. The behavior and control of the unit changes noticeably after applying a high emissivity coating with an increased premixed burner gas flow rate to the first cracking cell, cell 3, and reduced firing in the second cracking cell, cell 4. However, the total fuel consumed in the cracking cells remains constant before and after a high emissivity coating is applied. The main difference is the reduced firing in the last preheating cell, cell 2. Even on a pilot scale the application of a high emissivity coating results in fundamental changes to the process control. There results confirm that in order to properly evaluate energy savings, the entire process has to be examined, not limiting the observation to the cracking cells.

### 4.3. Tube-in-Box Radiative Heat Transfer Simulations

Based on spectral normal emissivity measurements, applying a high emissivity coating offers a way to increase the surface emissive properties. Detailed process simulations from scientific literature combined with the experimental results of the previous section suggest that the emissive properties have an impact on the firebox efficiency and the fuel firing rate. However, is it possible to quantitatively predict this effect without the need for an extensive three-dimensional Computational Fluid Dynamics simulation? The remaining part of this work will focus on the role of heat sink (reactor coil) emissive properties and enclosing adiabatic surface (furnace refractory) emissive properties when modelling radiative heat transfer.

Based on the pilot unit experiments, a reactor tube wall temperature, $T_{1}$, of 1173 K will be used as heat sink boundary condition for the tube-in-box model. For the initial simulations, conditions as shown in Figure 4a are used. A constant gas phase temperature, $T_{G}$, of 1373 K is assumed and simulations are performed using different emissive properties $\varepsilon_{1}$ and $\varepsilon_{2}$. In the next simulations, conditions are as shown in Figure 4b. The gas phase temperature, $T_{G}$, is increased to realize the same net heat flux, Q, to the inner tube, again using different boundary emissive properties $\varepsilon_{1}$ and $\varepsilon_{2}$.

#### 4.3.1. Gray-Plus-Clear Gas Simulation Results

**Figure 12:**
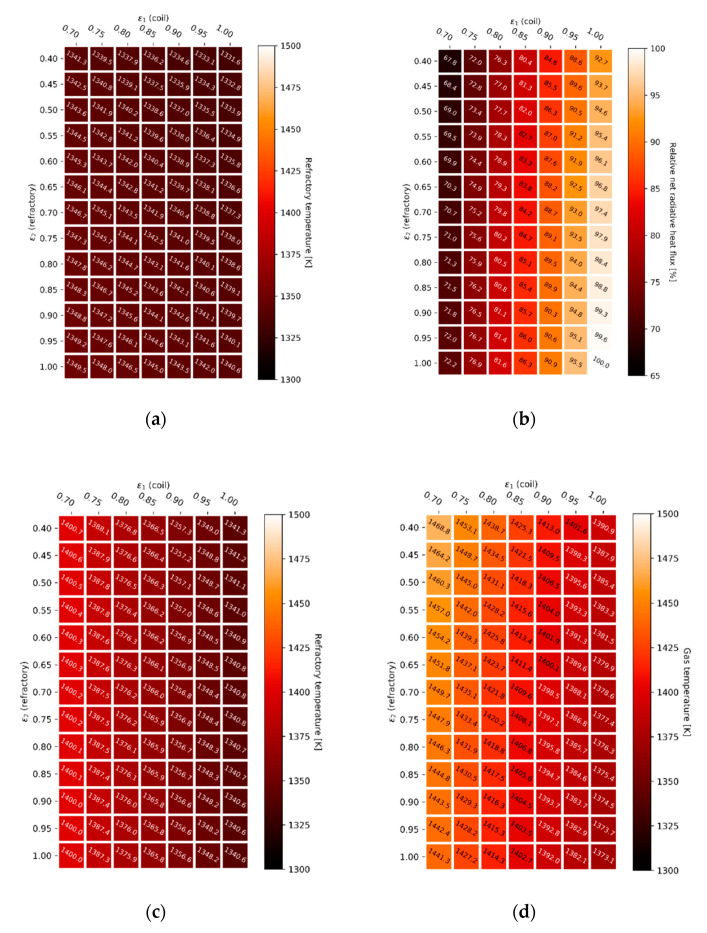
Refractory wall temperature (**a**) and radiative heat flux (**b**) for a constant gas phase temperature. Refractory wall temperature (**c**) and gas phase temperature (**d**) for a constant radiative heat flux in the presence of a gray-plus-clear gas.

In order to solve the tube-in-box radiative heat transfer problem, the total exchange area approach can be applied. The reactor tube emissivity, $\varepsilon_{1}$, is varied from 0.7 to 1.0 (x-axis) and the refractory emissivity, $\varepsilon_{2}$, is varied from 0.4 to 1.0 (y-axis) to give an almost complete overview of the effect of the emissive properties of the boundary surfaces on the radiative heat transfer inside the geometry. Calculated refractory wall temperatures are shown in Figure 12 for both the constant gas phase temperature simulations, Figure 12a, and for the constant heat flux simulations, Figure 12c, using a gray-plus-clear gas model. The heat maps showing the net radiative heat flux for a constant gas phase temperature, Figure 12b, and the gas phase temperature required to get a constant net heat flux, Figure 12d, complete Figure 12.

Keeping the gas phase temperature constant and changing the boundary emissive properties appears to have a notable effect on the net radiative heat flux towards the heat sink, shown in Figure 12b. A higher tube wall emissivity, $\varepsilon_{1}$, implies that more energy will be absorbed. This is clearly visualized by the last resistance before the heat sink in the “electric circuit analogy”, discussed in the supporting information. A higher boundary wall emissivity, $\varepsilon_{2}$, also results in an increase in net radiative heat flux towards the heat sink. An uncoated furnace wall with a higher probability of reflecting the incoming radiation, tends to reflect the radiation in an absorption band where it is re-absorbed by the gas, shown in Figure 7. A higher emissivity material, e.g., a high emissivity coating, will increase the probability that the incoming energy is re-emitted in a transparent window. Radiation will be more likely reach the heat sink in a transparent window and thus heat the load. The effect on the refractory wall temperature is negligible when keeping the gas phase temperature constant, as shown in Figure 12a. However, the refractory wall temperature, Figure 12c, increases noticeably when increasing the gas phase temperature to get the same net heat flux towards the process gas. It is important to emphasize the biquadratic relation between temperature and emitted radiation. As can be seen when increasing the gas phase temperature in Figure 12d, only a relatively minor increase is required to compensate for the offset caused when the boundary emissive properties are changed. Additionally, a change in gas phase temperature does not immediately translate into a major shift in firing rate. The gas phase temperature has to be increased with 95.7 K to offset a 32.2% decrease in net heat flux when lowering both the refractory wall emissivity, $\varepsilon_{2}$, from 1.0 to 0.7, and the radiant coil emissivity, $\varepsilon_{1}$, from 1.0 to 0.4 (difference between best case, high heat sink emissivity, and high adiabatic wall emissivity, and worst case-scenario, low heat sink emissivity and low adiabatic wall emissivity).

**Figure 13:**
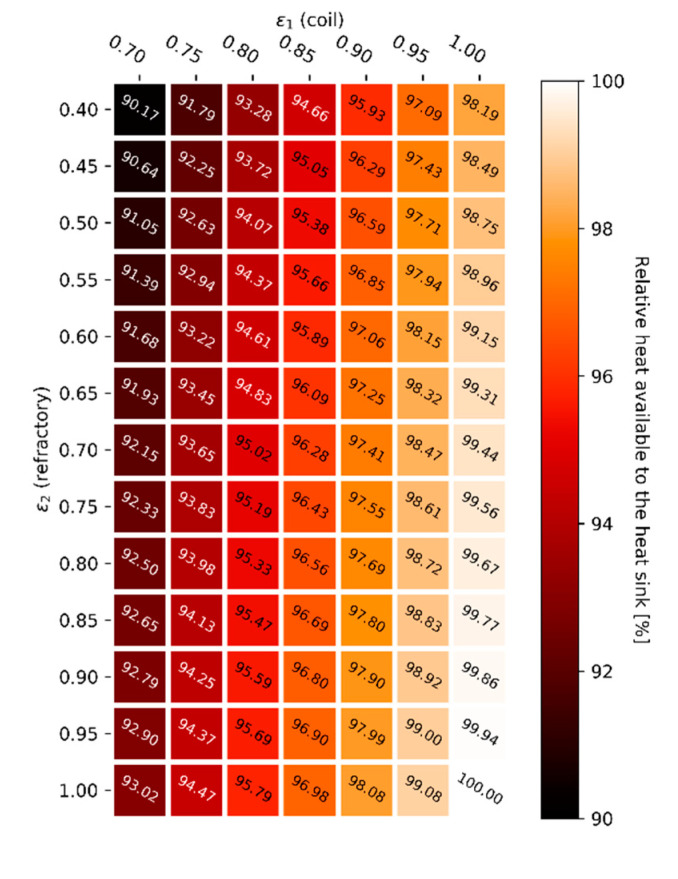
Relative heat available to the heat sink for a constant radiative heat flux in the presence of a gray-plus-clear gas.

To correlate the gas phase temperature, shown in Figure 12d when a constant heat flux is imposed, with an energy efficiency or firing rate, a complete furnace modelling approach including a combustion model would be required. In other words, a complete Computational Fluid Dynamics framework would be necessary to translate gas phase temperatures into a firing rate. This would considerably increase the computational cost. For the two-dimensional tube-in-box model the energy available to heat up the furnace load is determined by the enthalpy of combustion of methane minus the heat required to heat up the combustion gases to the equilibrium temperature. When the flue gas temperature is higher, less heat of combustion is available to the heat sink. Figure 13 shows the relative amount of energy available to heat the heat sink for the gray-plus-clear gas simulations with a constant heat flux, by translating the gas phase temperatures mentioned in Figure 12d to an energy efficiency using the previously described approach.

The main drawback when using a general weighted sum of gray gases approach, or the derived gray-plus-clear model, is that the band assumption is implicit. The radiative transfer equations are solved with a specific portion of the radiation transferred in the absorption bands. This portion, a, is calculated based on the gas phase temperature. In reality, radiative heat transfer is a bit more complicated as the fraction of total radiation transferred in a specific band also depends on the heat sink temperature and the surrounding adiabatic surface temperature. For this reason, the case study could be repeated with an exponential wide band modelling approach for the radiative transfer equation. The radiative transfer equation would in this case be solved in four absorption bands and five clear bands [5,24,25,53]. Since the exponential wide band model provides explicit upper and lower band limits, it would be possible to visualize the exact amount of radiation emitted and reflected by a surface in a specific band. These results visualize that in case a high emissivity coating is applied on the refractory wall, more radiation gets redistributed into the clear bands. As a result, the net heat flux to the coil increases when applying a high emissivity coating due to this redistribution of radiation into the clear bands.

#### 4.3.2. Gray Gas Simulation Results

**Figure 14:**
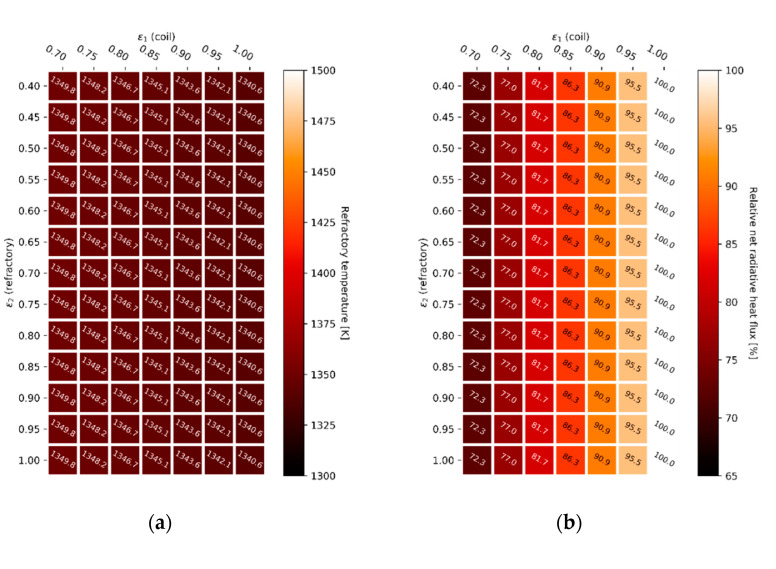

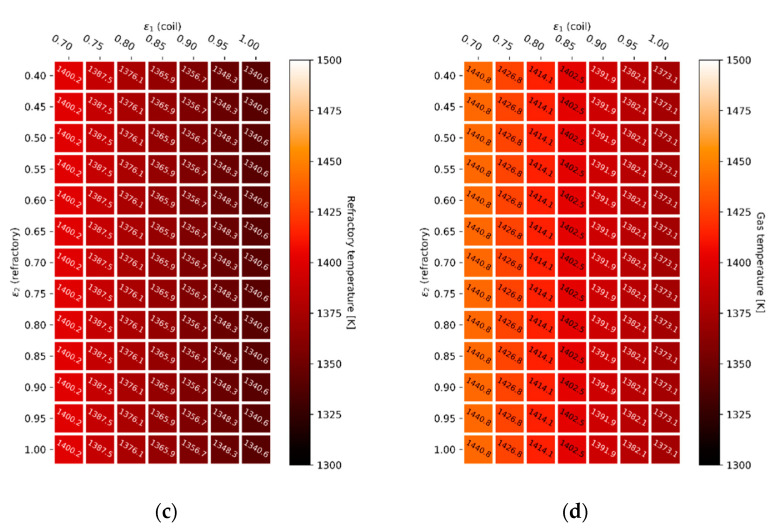
Refractory wall temperature (**a**) and radiative heat flux (**b**) for a constant gas phase temperature. Refractory wall temperature (**c**) and gas phase temperature (**d**) for a constant radiative heat flux in the presence of a gray gas.

Figure 14 shows the results for the tube-in-box heat transfer simulations using a gray gas model. This time the adiabatic surface emissivity, $\varepsilon_{2}$, plays no role in the radiative heat transfer. On the other hand, the effect of the heat sink emissivity, $\varepsilon_{1}$, remains visible when using a gray gas model. Again, a higher tube wall emissivity, $\varepsilon_{1}$, implies that more energy will be absorbed by the heat sink. Analogously, if the enclosing surface is not completely adiabatic but accounts for some heat losses, it is undesired to have a high furnace wall emissivity, $\varepsilon_{2}$, when using a gray gas model. In this case, the resistance in the “electric circuit analogy” should be maximized, in other words the reflectivity should be maximized to prevent heat losses through the refractory wall to the surroundings. However, in the case of a perfectly insulated enclosing surface, the gray gas model solutions are independent of the adiabatic wall emissivity, $\varepsilon_{2}$, as shown in Equation (10). With the gray gas approximation, all radiation striking the enclosing adiabatic surface will either be reflected or absorbed and re-emitted. Since there is no clear distinction between the reflected radiation, on the one hand, and the absorbed and re-emitted radiation, on the other hand, the enclosing surface can be regarded as a blackbody, whatever its emissivity is. The enclosing surface redirects all radiation towards the heat sink, which is by definition the fundamental property of a blackbody. A non-gray gas model is needed to distinguish between reflected and re-emitted radiation [27].

Overall, radiative transfer characteristics of an opaque wall can often be described with sufficient accuracy by gray emission, absorption, and reflection. However, the radiative properties of a molecular gas vary strongly and rapidly across the wavelength spectrum. As a consequence, the gray gas assumption almost never suffices [34,54]. A non-gray gas modelling technique is required to accurately capture the radiative heat transfer characteristics of the gas. Even though the emissive properties of a high emissivity coating can often be described as a gray surface, a non-gray modelling approach is necessary to simulate the effect of the enclosing wall as a redistributor of radiation.

## 5. Conclusions

There is a clear linear correlation between incident or outgoing radiation and surface emissivity. The origin of high emissivity coatings is based on this fundamental principle, which allows space vehicles to dissipate more radiation to the surroundings by increasing the surface emissive properties. Analogously, high emissivity coatings have also been making their way into the chemical industry for emitting surfaces. For a heat sink, namely a surface envisioned to absorb radiation, increasing the emissive properties allows for energy savings. This way, the incident radiation can be lowered to maintain the same net heat flux to the heat sink, e.g., lower firing rate when increasing the steam cracking coil emissivity. However, this argument has long been used against the application of high emissivity coatings on refractory walls. Refractory material is by nature reflective in the infrared wavelength region, so it is not inclined to absorb radiation which would result in undesirable heat losses to the outside surroundings [20]. However, when accounting for the absorption-emission spectra of flue gases, the opportunity of high emissivity coatings to absorb radiation with the possibility of re-emitting in a clear, non-absorbing window, makes research in the application of high emissivity coatings on refractory worthwhile. With a total normal emissivity of 0.79 for the high emissivity coating compared to 0.67 for the uncoated refractory, at 873 K, applying a high emissivity coating is found to significantly improve the emissive properties of a refractory surface. Steam cracking pilot unit experiments report a 5% reduction in firing rate after applying a high emissivity coating on the refractory wall. Radiative heat transfer simulations using a tube-in-box model show that the emissive properties of an adiabatic enclosing surface do not influence the temperature distribution in the presence of a gray gas. All energy is redirected towards the heat sink (tube) with the surrounding surface (box) acting as a blackbody. However, the gray-plus-clear gas model confirms that the furnace can be operated at a lower temperature when the emissive properties of an adiabatic surface are improved. Energy radiated by the hot flue gas is more likely to be absorbed by a surface and consequently re-emitted by that surface in a clear gas window when its emissive properties are maximized, resulting in energy savings.

The radiative heat transfer simulations show that the possible gains in terms of efficiency are a matter of percentages when changing the boundary wall emissive properties. This makes it statistically challenging to experimentally evaluate the impact on a pilot or industrial scale. In that case, Computational Fluid Dynamics simulations, preferably with a spectral radiation model, are the preferred option to accurately evaluate possible changes to the process after coating the reactor coil or the furnace wall. Changing the radiant coil emissive properties is expected to have the most impact on the overall process since proportionally more heat is absorbed. In this case, changing the emissive properties has a direct effect, not a secondary effect of redistributing radiation, which is the case when coating the refractory wall. However, similar to the lack of experimental data related to the emissive properties of refractory materials, more information should be gathered to quantify the difference in emissive properties between uncoated and coated high-temperature alloys.

## Figures and Tables

**Table 1:** Energy savings after applying a high emissivity coating on the furnace refractory reported in literature.

Reference	Year	Type of Furnace	Energy Savings
Hellander et al. [28]	1991	Annealing furnace	17%
Benko et al. [29]	2002	Brick tunnel kiln	6%
Adams et al. [26]	2015	Glass melting furnace	8%
Adams et al. [26]	2015	Ethylene dichloride furnace	5%
Adams et al. [26]	2015	Steam cracking furnace	6%

**Table 2:** Operating conditions for the steam cracking experiments.

Procedure	Duration	Temperature [K]		Feed Flow
	[h]	Cell 3	Cell 4	[-]
**Steam Treatment**	12	1073	1073	2 kg/h H_2_O
**Pre-Sulfiding**	3	1008	1143	4 kg/h H_2_O, 300 ppm-w S/H_2_O
**Steam Cracking**	6	1013	1111	10 kg/h C_3_H_8_, 4 kg/h H_2_O, 100 ppm-w S/HC
**Decoking**	-	-	-	-
**Step 1**	±1	1073	1073	1 kg/h H_2_O, 1 kg/h air
**Step 2**	±1	1173	1173	1 kg/h H_2_O, 1 kg/h air
**Step 3**	±1	1173	1173	1 kg/h air

**Table 3:** Natural gas flow rate to the pilot unit at steady state for the different experiments.

Case	Natural Gas Flow Rate [m^3^/h]
uncoated 1	7.6
uncoated 2	7.5
coated 1	7.1
coated 2	7.2

## Data Availability

Data sharing not applicable.

## Supplementary Materials

The following are available online at https://www.mdpi.com/1996-1944/14/4/880/s1, Figure S1: Electric circuit analogy of a radiative heat transfer problem involving two gray surfaces, Figure S2: Electric circuit analogy for a configuration with one sink surface and an adiabatic refractory sur-face.
