# Supplementary material for: The Effect of Refractory Wall Emissivity on the Energy Efficiency of a Gas-Fired Steam Cracking Pilot Unit
Source: Materials (Basel). 2021 Feb 12;14(4):880. doi: 10.3390/ma14040880 (PMC7918708; doi:10.3390/ma14040880)
Supplement: Supplementary file 1 [file materials-14-00880-s001.zip › materials-1074570-supplementary.pdf]

materials MDPI

Supplementary

The Effect of Refractory Wall Emissivity on the Energy Efficiency of a Gas-Fired Steam Cracking Pilot Unit

Stijn Vangaever [1], Joost Van Thielen [2], Jeremy Hood [3], John Olver [3], Petra Honnerovà [4], Geraldine J. Heynderickx [1] and Kevin M. Van Geem [1,*]

1 Laboratory for Chemical Technology, Ghent University, Technologiepark 125, 9052 Ghent, Belgium; Stijn.Vangaever@UGent.be (S.V.); Geraldine.Heynderickx@UGent.be (G.J.H.)
2 CRESS B.V., Deltahoek 34, 4511 PA Breskens, The Netherlands; Joost.VanThielen@CRESSbv.nl
3 Emisshield Inc., 2000 Kraft Drive VA 24060, Blacksburg, VA, USA; Jeremy.Hood@Emisshield.com (J.H.); John.Olver@Emisshield.com (J.O.)
4 New Technologies Research Centre, University of West Bohemia, Univerzitní 8, 306 14 Pilsen, Czech Republic; petrahon@ntc.zcu.cz
* Correspondence: Kevin.VanGeem@UGent.be

Note: The content of the supporting information is based on dedicated radiative heat transfer textbooks [1–3]. The following equations intend to provide the reader with the necessary background required to derive/understand the formulas used in the corresponding paper.

1. Radiative Heat Transfer Between Black Surfaces

The net radiative energy interchange, Q_{ij}, between two black surfaces, i and j, with surface areas, A_i and A_j, is calculated as:

$$Q_{ij} = A_i F_{ij} E_i - A_j F_{ji} E_j \tag{S1}$$

where the total energy E_i leaving a blackbody surface i is determined by the Stefan–Boltzmann law. In order to introduce symmetry, the concept of direct surface-to-surface exchange area, $\overline{s_i s_j}$, for finite black surfaces is introduced:

$$\overline{s_i s_j} = A_i F_{ij} \tag{S2}$$

where the view factor, F_{ij}, is equivalent to the portion of radiation leaving surface i and directly impacting surface j. Because of the reciprocity relation $A_i F_{ij} = A_j F_{ji} \Leftrightarrow \overline{s_i s_j} = \overline{s_j s_i}$, the expression for the net radiative energy exchange between two finite black surfaces, Equation (S1), simplifies to:

$$Q_{ij} = \overline{s_i s_j}(E_i - E_j) \tag{S3}$$

The formal definition of the direct surface-to-surface exchange area is given by:

$$\overline{s_i s_j} = \oiint_{A_i} \oiint_{A_j} \frac{cos\varphi_i cos\varphi_j}{\pi r^2} dA_j dA_i \tag{S4}$$

The direct surface-to-surface exchange area can be calculated using Equation (S2) when view factors are known. View factors between surfaces in simplified geometries are often described in literature or can be derived analytically. For more complex geometries they are often calculated using Monte Carlo techniques.

2. Radiative Heat Transfer Between Gray Surfaces

For gray surfaces an emission coefficient, ε, is introduced. This general "emissivity" corresponds to the total hemispherical emissivity previously introduced in Equation (6).

When a surface is in thermodynamic equilibrium with the surroundings, all absorbed radiation will be re-emitted, according to Kirchhoff's law of thermal radiation. For a gray surface, the total radiative flux leaving a surface is defined as the radiosity, J. In other words, the radiosity is made up by two contributions: the radiation emitted by the surface on the one hand and the reflected radiation on the other [4].

$$J_i \equiv \varepsilon_i E_i + \rho_i H_i = \begin{pmatrix}\text{Radiation emitted}\\ \text{by surface } i\end{pmatrix} + \begin{pmatrix}\text{Radiation reflected}\\ \text{by surface } i\end{pmatrix} \quad \text{(S5)}$$

where the reflectivity, ρ, equals $1-\varepsilon$, as opaque bodies to not transmit radiation. The total incident radiation is expressed by H, while E corresponds to the energy emitted by a blackbody surface at the same temperature. Finally, the energy balance over a gray surface yields the following equation for the total heat supplied to that surface:

$$Q_i = A_i[J_i - H_i] = A_i\left[J_i - \frac{J_i - \varepsilon_i E_i}{1-\varepsilon_i}\right] = \frac{\varepsilon_i}{1-\varepsilon_i} A_i[E_i - J_i] \quad \text{(S6)}$$

The "electric circuit analogy", as shown in Figure S1 for a system with one heat source and one heat sink, helps to visualize radiative heat transfer.

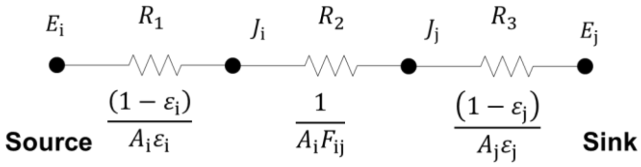

Figure S1. Electric circuit analogy of a radiative heat transfer problem involving two gray surfaces.

The net radiative exchange between these two gray surfaces can be interpreted as three resistances in series, based on the electric circuit analogy:

$$Q_{ij} = \frac{E_i - E_j}{R_1 + R_2 + R_3} = \frac{E_i - E_j}{\frac{(1-\varepsilon_i)}{A_i\varepsilon_i} + \frac{1}{A_i F_{ij}} + \frac{(1-\varepsilon_j)}{A_j\varepsilon_j}} \quad \text{(S7)}$$

Based on Equation (S7), the effect of the (changes in) emissivity on the radiative energy exchange between two gray surfaces becomes clearer. A higher emissivity reduces the "resistance", which implies that a lower driving force, $E_i - E_j$, is needed to realize the same radiative energy exchange between two gray surfaces [5].

For geometrically more complex systems involving several participating gray surfaces, the total surface-to-surface exchange area for each pair of surfaces, $\overline{S_iS_j}$, is introduced, in analogy to the direct surface-to-surface exchange area, $\overline{s_is_j}$, for blackbody surfaces. The net radiative heat transfer between two gray surfaces is expressed as:

$$Q_{ij} = \overline{S_iS_j}(E_i - E_j) \quad \text{(S8)}$$

This total surface-to-surface exchange area accounts for:

- The area of both surfaces.
- The shape, orientation and spacing of both surfaces.
- The radiative properties of both surfaces.
- The reflection caused by other additional surfaces.
- The possibility to account for participating media (discussed later).

All this information is combined by introducing the concept of total surface-to-surface exchange area. The main advantage of the total surface-to-surface exchange area is that the electric circuit analogy no longer needs to be visualized. Whereas the electric circuit analogy offers a rapid visualization for problems involving up to three surfaces, the method gets tedious once more surfaces are involved. The latter is bound to happen when modelling three dimensional problems. A general derivation of the total surface-to-surface exchange area in multi-surface problems is given in the following section.

3. Total Surface-to-Surface Exchange Area between Two Gray Surfaces

The total radiative flux leaving a surface, J_j, consists of the radiation emitted by the surface and multiple reflective contributions:

$$A_j J_j = A_j \varepsilon_j E_j + \rho_j \sum_i \overline{s_i s_j} J_i \tag{S9}$$

By introducing the Kronecker delta symbol, δ_{ij}, a shorthand notation is introduced:

$$\sum_i \left(\overline{s_i s_j} - \delta_{ij} A_j/\rho_j\right) J_i = -\frac{A_j \varepsilon_j}{\rho_j} E_j \tag{S10}$$

In matrix notation, this expression becomes:

$$\begin{bmatrix} \overline{s_1 s_1} - A_1/\rho_1 & \overline{s_1 s_2} & \overline{s_1 s_3} & \\ \overline{s_2 s_1} & \overline{s_2 s_2} - A_2/\rho_2 & \overline{s_2 s_3} & \cdots \\ \overline{s_3 s_1} & \overline{s_3 s_2} & \overline{s_3 s_3} - A_3/\rho_3 & \\ \vdots & \vdots & \vdots & \end{bmatrix} \cdot \begin{bmatrix} J_1 \\ J_2 \\ J_3 \\ \vdots \end{bmatrix} = \begin{bmatrix} -A_1 \varepsilon_1 E_1/\rho_1 \\ -A_2 \varepsilon_2 E_2/\rho_2 \\ -A_3 \varepsilon_3 E_3/\rho_3 \\ \vdots \end{bmatrix} \tag{S11}$$

For the net heat flux between two gray surfaces all emissive contributions in Equation (S11), except for the emitting surface i, are assigned the value of zero. Equation (S6) can be rewritten to account for the net flux into j with one sole emitting surface i [6]:

$$Q_{ij} = \frac{A_j \varepsilon_j}{\rho_j}\left(J_j^{(i)}/E_i - \delta_{ij}\varepsilon_i\right)\left[E_i - E_j\right] \tag{S12}$$

This gives the general expression for the total surface-to-surface exchange area, based on Equation (S8):

$$\overline{S_i S_j} = \frac{A_j \varepsilon_j}{\rho_j}\left(J_j^{(i)}/E_i - \delta_{ij}\varepsilon_i\right) \tag{S13}$$

where the superscript in the notation $J_j^{(i)}$ is used to stress by which surface, i, the radiation is emitted. Using Cramer's rule to solve Equation (S11) for $J_j^{(i)}$, a general expression can be obtained for the total surface-to-surface exchange area, $\overline{S_i S_j}$. The expression is based on the cofactor of the ith row and jth column of the square coefficient matrix in Equation (S11) (later referred to as M) and the determinant of the same coefficient matrix [6]. The matrix inversion required to calculate the determinant of the coefficient matrix makes it tedious to get an exact analytical solution for problems involving more than two radiating surfaces. For that reason, we will continue with a simplified geometry involving only two surfaces.

$$\begin{gathered} \begin{bmatrix} \overline{s_1 s_1} - A_1/\rho_1 & \overline{s_1 s_2} \\ \overline{s_1 s_2} & \overline{s_2 s_2} - A_2/\rho_2 \end{bmatrix} \cdot \begin{bmatrix} J_1^{(1)} \\ J_2^{(1)} \end{bmatrix} = \begin{bmatrix} -A_1 \varepsilon_1 E_1/\rho_1 \\ 0 \end{bmatrix} \Leftrightarrow \\ \begin{bmatrix} J_1^{(1)} \\ J_2^{(1)} \end{bmatrix} = \frac{1}{\det(M)} \begin{bmatrix} \overline{s_2 s_2} - A_2/\rho_2 & -\overline{s_1 s_2} \\ -\overline{s_1 s_2} & \overline{s_1 s_1} - A_1/\rho_1 \end{bmatrix} \cdot \begin{bmatrix} -A_1 \varepsilon_1 E_1/\rho_1 \\ 0 \end{bmatrix} \Leftrightarrow \\ \begin{bmatrix} J_1^{(1)} \\ J_2^{(1)} \end{bmatrix} = \frac{1}{\det(M)} \begin{bmatrix} (\overline{s_2 s_2} - A_2/\rho_2)(-A_1 \varepsilon_1 E_1/\rho_1) \\ \overline{s_1 s_2} A_1 \varepsilon_1 E_1/\rho_1 \end{bmatrix} \end{gathered} \tag{S14}$$

Substitution of $J_2^{(1)}$ in Equation (S13) results in an expression for the total surface-to-surface exchange area for the net radiative exchange between two gray surfaces:

$$\overline{S_1 S_2} = \frac{\varepsilon_1 A_1}{\rho_1}\left[\frac{\varepsilon_2 A_2}{\rho_2}\left(\frac{\overline{s_1 s_2}}{\det(M)}\right)\right] = \frac{1}{\dfrac{(1-\varepsilon_1)}{A_1 \varepsilon_1} + \dfrac{1}{A_1 F_{12}} + \dfrac{(1-\varepsilon_2)}{A_2 \varepsilon_2}} \tag{S15}$$

with the determinant for the coefficient matrix M:

$$\det(M) = \begin{vmatrix} \overline{s_1s_1} - A_1/\rho_1 & \overline{s_1s_2} \\ \overline{s_1s_2} & \overline{s_2s_2} - A_2/\rho_2 \end{vmatrix} = (\overline{s_1s_1} - A_1/\rho_1)(\overline{s_2s_2} - A_2/\rho_2) - (\overline{s_1s_2})^2 \quad \text{(S16)}$$

As expected, the total surface-to-surface exchange area matches the inverse of the three resistances in series shown in the electric circuit analogy in Figure S1.

4. Radiative Heat Transfer in the Presence of a Gray Gas

In the presence of a gray gas having an absorption coefficient, K, averaged over the entire spectrum, the direct surface-to-surface exchange area, previously introduced in Equation (S4), becomes:

$$\overline{s_is_j} = \oiint_{A_i} \oiint_{A_j} \frac{e^{-Kr}}{\pi r^2} cos\varphi_i cos\varphi_j dA_j dA_i \quad \text{(S17)}$$

and the direct gas-to-surface exchange area is introduced as:

$$\overline{s_ig_j} = \oiint_{A_i} \oiiint_{V_j} K \frac{e^{-Kr}}{\pi r^2} cos\varphi_i dV_j \, dA_i \quad \text{(S18)}$$

Solving the aforementioned integrals is time consuming. Additionally, the integration as to be reperformed every time the absorption coefficient changes, e.g., when the composition or the temperature of the participating gas changes. This is typically prevented by introducing the concept of the mean beam length, L. The transmissivity defined as $\tau = e^{-KL}$, becomes a constant value and can be placed out of the integral.

$$\overline{s_is_j} = e^{-KL} \oiint_{A_i} \oiint_{A_j} \frac{cos\varphi_i cos\varphi_j}{\pi r^2} dA_j dA_i = \tau A_i F_{ij} \quad \text{(S19)}$$

The electric circuit analogy for the tube-in-box problem, described in Figure 4, is visualized in Figure S2.

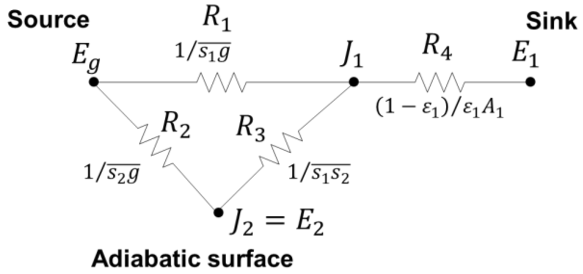

Figure S2. Electric circuit analogy for a configuration with one sink surface and an adiabatic refractory surface.

The direct exchange areas for the electric circuit analogy, shown in Figure S2, are given by:

$$\begin{cases} \overline{s_1s_1} = A_1F_{11}\tau \\ \overline{s_1s_2} = A_1F_{12}\tau \\ \overline{s_2s_2} = A_2F_{22}\tau \\ \overline{s_1g} = (A_1F_{11} + A_1F_{12})(1-\tau) \\ \overline{s_2g} = (A_2F_{21} + A_2F_{22})(1-\tau) \end{cases} \quad \text{(S20)}$$

For the simplified tube-in-box model, the primary example in the corresponding paper, the direct exchange areas can be simplified as a consequence of the symmetry of the problem. As the central tube is convex, the view factor matrix simplifies to:

$$F = \begin{bmatrix} F_{11} & F_{12} \\ F_{21} & F_{22} \end{bmatrix} = \begin{bmatrix} 0 & 1 \\ A_1/A_2 & 1 - A_1/A_2 \end{bmatrix} \quad \text{(S21)}$$

By substituting the view factors of the tube-in-box model into the general formulation of the direct exchange areas for two surfaces, Equation (S21) can be simplified to:

$$\begin{cases} \overline{s_1 s_1} = 0 \\ \overline{s_1 s_2} = A_1 \tau \\ \overline{s_2 s_2} = A_2 (1 - A_1/A_2)\tau \\ \overline{s_1 g} = A_1 (1 - \tau) \\ \overline{s_2 g} = A_2 (1 - \tau) \end{cases} \tag{S22}$$

The total resistance of the electric circuit corresponding to the tube-in-box problem, visualized in Figure S2, gives:

$$Q_{G1,tot} = \frac{E_g - E_1}{R_{tot}} \text{ with } R_{tot} = \frac{1}{\left(\frac{1}{R_1}\right) + \left(\frac{1}{R_2 + R_3}\right)} + R_4 \tag{S23}$$

$$\Leftrightarrow R_{tot} = \frac{1}{\frac{1}{A_1(1-\tau)} + \frac{1}{A_2(1-\tau) + A_1\tau}} + (1 - \varepsilon_1)/\varepsilon_1 A_1$$

The electric circuit where energy is transferred from the gas to the reactor tube (from source to sink) consists of four resistances. Energy is either transferred directly from the gas to the coil, determined by R_2, or is transferred from the gas to the refractory wall and consequently to the coil, corresponding to two resistances in series, R_2 and R_3. These two options, on the one hand directly from gas to coil or on the other hand from gas to coil through reflection or absorption and re-emission of the refractory wall, are regarded as two resistances in parallel. One final resistance, R_4, is determined by the receiving surface emissivity, ε_1.

5. Total Surface-to-Surface Exchange Area in the Presence of a Gray Gas

In analogy to Equation (S9), the total incoming radiation in the presence of a participating gas can be expressed as:

$$A_i \frac{J_i - \varepsilon_i E_i}{\rho_i} = \sum_j J_j \overline{s_j s_i} + \overline{g s_i} E_g \tag{S24}$$

which transforms into the following matrix for a two-surface problem in the presence of a gray gas:

$$\begin{bmatrix} \overline{s_1 s_1} - A_1/\rho_1 & \overline{s_1 s_2} \\ \overline{s_1 s_2} & \overline{s_2 s_2} - A_2/\rho_2 \end{bmatrix} \cdot \begin{bmatrix} J_1 \\ J_2 \end{bmatrix} = \begin{bmatrix} -A_1\varepsilon_1 E_1/\rho_1 - \overline{g s_1} E_g \\ -A_2 \varepsilon_2 E_2/\rho_2 - \overline{g s_2} E_g \end{bmatrix} \tag{S25}$$

The total surface-to-surface and gas-to-surface exchange areas are derived in analogy to Equations (S13) and (S14).

$$\begin{aligned} \overline{S_1 S_2} &= \frac{A_1 \varepsilon_1 \varepsilon_2 F_{12}}{1/\tau + \tau\rho_1\rho_2(1 - F_{12}/C_2) - \rho_1(1 - F_{12}) - \rho_2(1 - F_{21})} \\ \overline{S_1 S_1} &= \frac{A_1 \varepsilon_1^2 \left(F_{11} + \rho_2 \tau (F_{12}/C_2 - 1)\right)}{1/\tau + \tau\rho_1\rho_2(1 - F_{12}/C_2) - \rho_1(1 - F_{12}) - \rho_2(1 - F_{21})} \\ \overline{S_2 S_2} &= \frac{A_2 \varepsilon_2^2 \left(F_{22} + \rho_1 \tau (F_{12}/C_2 - 1)\right)}{1/\tau + \tau\rho_1\rho_2(1 - F_{12}/C_2) - \rho_1(1 - F_{12}) - \rho_2(1 - F_{21})} \\ \overline{G S_1} &= \frac{A_1 \varepsilon_1 (1-\tau)\left(1/\tau + \rho_2 (F_{12}/C_2 - 1)\right)}{1/\tau + \tau\rho_1\rho_2(1 - F_{12}/C_2) - \rho_1(1 - F_{12}) - \rho_2(1 - F_{21})} \\ \overline{G S_2} &= \frac{A_2 \varepsilon_2 (1-\tau)\left(1/\tau + \rho_1 (F_{12}/C_2 - 1)\right)}{1/\tau + \tau\rho_1\rho_2(1 - F_{12}/C_2) - \rho_1(1 - F_{12}) - \rho_2(1 - F_{21})} \end{aligned} \tag{S26}$$

with $C_2 = A_2/A_{tot}$ and $A_{tot} = A_1 + A_2$.

Substitution of the view factor matrix for the tube-in-box model, Equation (S21), in the aforementioned Equation (S26), results in the final total exchange areas for the tube-in-box geometry:

$$\overline{S_1S_2} = \frac{A_1\varepsilon_1\varepsilon_2}{D}$$
$$\overline{GS_1} = \frac{A_1\varepsilon_1\varepsilon_G(1/\tau + \rho_2\, A_1/A_2)}{D}$$
$$\overline{GS_2} = \frac{A_2\varepsilon_2\varepsilon_G(1/\tau + \rho_1\, A_1/A_2)}{D} \qquad \text{(S27)}$$
$$\overline{GS_2} = \frac{A_2\varepsilon_2\varepsilon_G(1/\tau + \rho_1\, A_1/A_2)}{D}$$

$$\text{with } D = 1/\tau - \rho_2\left[1 - \frac{A_1}{A_2}(1 - \tau\rho_1)\right]$$

In the tube-in-box model, the enclosing surface is assumed to be adiabatic, indicating that all incoming energy will have to be redirected:

$$Q_{G1,tot} = \overline{GS_1}(E_g - E_1) + \overline{GS_2}(E_g - E_2) = \left(\overline{GS_1} + \frac{1}{\frac{1}{\overline{GS_2}} + \frac{1}{\overline{S_1S_2}}}\right)(E_g - E_1) \qquad \text{(S28)}$$
$$\text{since } \overline{GS_2}(E_g - E_2) = \overline{S_1S_2}(E_2 - E_1) \Leftrightarrow E_2 = \frac{\overline{GS_2}E_g + \overline{S_1S_2}E_1}{\overline{S_1S_2} + \overline{GS_2}}$$

This total surface-to-surface exchange area solution proves to be a valuable alternative to the electric circuit approach when more surfaces are present.

6. Total Surface-to-Surface Exchange Area in the Presence of a Gray-Plus-Clear Gas

A radiating gas departs from grayness in two ways [7].

- Its transmittance τ along successive path lengths due to surface reflection keeps increasing, while it remains constant for a gray gas. At wavelengths with high absorption the incremental absorption decreases with increasing path length.
- Gas emissivity, ε_G, and gas absorptivity, α_G, have different values unless the gas temperature equals the temperature of the radiating surface.

For a gas and an enclosing black surface, the concept of equivalent gray gas emissivity is introduced to circumvent this issue:

$$Q_{GS_1} = \varepsilon_G E_G - \alpha_G E_1 \equiv \varepsilon_{G,e}(E_G - E_1) \text{ with } \varepsilon_{G,e} = \frac{\varepsilon_G E_G - \alpha_G E_1}{E_G - E_1} \qquad \text{(S29)}$$

In the weighted sum of gray gasses model, the gas emissivity, ε_G, is expressed as a weighted sum of gray gas emissivities in (n + 1) wavelength bands:

$$\varepsilon_G = \sum_0^n a_i\varepsilon_{G,i} = \sum_0^n a_i(1 - e^{-K_iL}) \text{ with } \sum\nolimits_0^n a_i = 1 \qquad \text{(S30)}$$

In the following calculations, the gray-plus-clear simplification is used. Introducing one clear and one absorption band, Equation (S30) becomes:

$$\varepsilon_G(L) = a_1(1 - e^{-K_1L}) + a_2(1 - e^{-K_2L}) \qquad \text{(S31)}$$

with $K_1 = 0$ and $a_2 \neq 0$, to satisfy the one clear and one absorption band assumption. Equation (S31) simplifies to:

$$\varepsilon_G(L) = a(1 - e^{-KL}) = a(1 - \tau) \qquad \text{(S32)}$$

The parameter a can be determined by calculating the gas phase absorptivity, ε_G, for two different path lengths.

$$\begin{cases} \varepsilon_G(L) = a(1-\tau) \\ \varepsilon_G(2L) = a(1-e^{-2KL}) = a(1-\tau^2) = a(1-\tau)(1+\tau) = \varepsilon_G(L)(1+\tau) \end{cases} \tag{S33}$$

In this set of equations, the transmissivity, τ, and coefficient, a, representing the total energy fraction of the blackbody energy distribution emitted in the non-clear absorption band, are the only two unknowns.

The electric circuit analogy is no longer valid when working with non-gray gasses. There is no longer "one type of current", since an emitting surface emits partly in the clear window and partly in one of the absorbing wavelength bands. For the gray-plus-clear gas assumption, all absorbing wavelength bands are lumped, resulting in just one transparent and one absorbing wavelength band. The total surface-to-surface and gas-to-surface exchange areas are then calculated from:

$$\begin{aligned} \overline{S_1S_2}' &= (1-a)\frac{A_1\varepsilon_1\varepsilon_2F_{12}}{1+\rho_1\rho_2(1-F_{12}/C_2)-\rho_1(1-F_{12})-\rho_2(1-F_{21})} \\ &\quad + a\frac{A_1\varepsilon_1\varepsilon_2F_{12}}{1/\tau+\tau\rho_1\rho_2(1-F_{12}/C_2)-\rho_1(1-F_{12})-\rho_2(1-F_{21})} \\ \overline{GS_1}' &= \frac{A_1\varepsilon_1\varepsilon_{G,e}\big(1/\tau+\rho_2(F_{12}/C_2-1)\big)}{1/\tau+\tau\rho_1\rho_2(1-F_{12}/C_2)-\rho_1(1-F_{12})-\rho_2(1-F_{21})} \\ \overline{GS_2}' &= \frac{A_2\varepsilon_2\varepsilon_{G,e}\big(1/\tau+\rho_1(F_{12}/C_2-1)\big)}{1/\tau+\tau\rho_1\rho_2(1-F_{12}/C_2)-\rho_1(1-F_{12})-\rho_2(1-F_{21})} \end{aligned} \tag{S34}$$

hereby accounting for the corrected transmissivity in the absorption band $\tau = 1-\varepsilon_{G,e}/a$

When the view factors of the tube-in-box model, Equation (S21), are taken into account the total exchange areas are calculated as:

$$\begin{aligned} \overline{S_1S_2}' &= (1-a)\frac{A_1\varepsilon_1\varepsilon_2}{1-\rho_2(1-\varepsilon_1A_1/A_2)} + a\frac{A_1\varepsilon_1\varepsilon_2}{D} \\ \overline{GS_1}' &= \frac{A_1\varepsilon_1\varepsilon_{G,e}(1/\tau+\rho_2A_1/A_2)}{D} \\ \overline{GS_2}' &= \frac{A_2\varepsilon_2\varepsilon_{G,e}(1/\tau+\rho_1A_1/A_2)}{D} \\ \text{with } D &= 1/\tau-\rho_2\left[1-\frac{A_1}{A_2}(1-\tau\rho_1)\right] \end{aligned} \tag{S35}$$

Gray gas and gray-plus-clear gas radiative heat transfer simulations are performed in the corresponding paper for the tube-in-box demonstration case. The goal is to highlight the effect of the boundary emissive properties, ε_1 and ε_2, on the radiative heat transfer from a participating gas to a central heat sink. The effect of the boundary wall emissive properties on the radiative heat transfer is far from understood and this approach aims to show the working principle behind high emissivity coatings. The conclusion is that a gray gas model suffices to capture the heat sink behavior of a reactor coil, but a non-gray gas model, that is able to account for the absorption and re-emission in specific bands, is necessary to accurately model the benefits of applying a high emissivity coating on the furnace wall. A high emissivity coating on a refractory wall increases the probability of absorbing radiation in a non-clear window and consequently re-emitting the radiation in a clear window where the probability increases to reach the heat sink.

References

1. Perry, R.; Green, D.; Maloney, *J., Perry's chemical engineers handbook*, 7th Int. Ed.; McGraw Hill: New York, NY, USA, 1998.
2. Green, D.W.; Perry, R.H., *Perry's Chemical Engineers' Handbook*, 8th ed.; McGraw-Hill Education: New York, NY, USA, 2007.
3. Avallone, E.A.; Baumeister, T.; Steidel, R.F. Marks' Standard Handbook for Mechanical Engineers, 9th Edition. *J. Eng. Ind.* **1991**, *113*, 118–119, doi:10.1115/1.2899615.
4. Modest, M.F., *Radiative heat transfer*, 3rd ed.; Academic press: Cambridge, MA, United States, 2013.
5. Cengel, Y.A.; Pérez, H., *Heat transfer: A practical approach. Transferencia de calor*, Mc Graw Hill: Mexico 2004.
6. Hottel, H.C.; Sarofim, A.F., *Radiative Transfer*. McGraw-Hill: New York, NY, USA, 1967.
7. Perry, R.H.; Green, D.W.; Maloney, J., *Perry's Chemical Engineers' Handbook*; McGraw-Hill: New York, NY, USA 1997.
